# Cigarette Smoke Condensate Exposure Changes RNA Content of Extracellular Vesicles Released from Small Airway Epithelial Cells

**DOI:** 10.3390/cells8121652

**Published:** 2019-12-17

**Authors:** Tiziana Corsello, Andrzej S. Kudlicki, Roberto P. Garofalo, Antonella Casola

**Affiliations:** 1 Department of Pediatrics, Division of Clinical and Experimental Immunology and Infectious Disease (CEIID), The University of Texas Medical Branch at Galveston (UTMB), Galveston, TX 77555, USA; ticorsel@utmb.edu (T.C.); rpgarofa@utmb.edu (R.P.G.); 2 Institute for Translational Sciences, UTMB, Galveston, TX 77555, USA; askudlic@utmb.edu; 3 Department of Biochemistry and Molecular Biology, UTMB, Galveston, TX 77555, USA; 4 Department of Microbiology and Immunology, UTMB, Galveston, TX 77555, USA; 5 Department of Pharmacology and Toxicology, UTMB, Galveston, TX 77555, USA

**Keywords:** airway epithelial cells, microvesicles, miRNAs, secondhand smoke, next generation sequencing

## Abstract

Exposure to environmental tobacco smoke (ETS) is a known risk factor for the development of chronic lung diseases, cancer, and the exacerbation of viral infections. Extracellular vesicles (EVs) have been identified as novel mediators of cell–cell communication through the release of biological content. Few studies have investigated the composition/function of EVs derived from human airway epithelial cells (AECs) exposed to cigarette smoke condensate (CSC), as surrogates for ETS. Using novel high-throughput technologies, we identified a diverse range of small noncoding RNAs (sncRNAs), including microRNA (miRNAs), Piwi-interacting RNA (piRNAs), and transfer RNA (tRNAs) in EVs from control and CSC-treated SAE cells. CSC treatment resulted in significant changes in the EV content of miRNAs. A total of 289 miRNAs were identified, with five being significantly upregulated and three downregulated in CSC EVs. A total of 62 piRNAs were also detected in our EV preparations, with five significantly downregulated and two upregulated in CSC EVs. We used TargetScan and Gene Ontology (GO) analysis to predict the biological targets of hsa-miR-3913-5p, the most represented miRNA in CSC EVs. Understanding fingerprint molecules in EVs will increase our knowledge of the relationship between ETS exposure and lung disease, and might identify potential molecular targets for future treatments.

## 1. Introduction

Environmental tobacco smoke (ETS) exposure is associated with an increased frequency of lower respiratory tract infections, increased incidence and severity of asthma episodes, overall decreased pulmonary function, and cancer development.

Extracellular vesicles (EVs) are membrane particles released by virtually all cells, shuttling active biological molecules such as proteins, lipids, and nucleic acids to neighboring cells, and to distant sites [1,2,3]. EVs have been isolated from various biofluids such as blood, breast milk, bronchial lavage, saliva, urine, amniotic, and cerebrospinal fluids [4,5,6,7,8,9]. EVs represent a heterogeneous population and vary in size (30–2000 nm in diameter) and composition, based on the cellular origin and environmental stimuli [10,11,12]. According to their diameter, EVs can be classified into two general subgroups: 1) small EVs (<200nm), and 2) medium/large EVs (>200 nm) [10]. Recent studies have identified EVs as critical players in intercellular communication under various physiological and pathological conditions such as neurodegenerative diseases, cancer, preterm birth, angiogenesis, immune responses, and viral infections [3,13,14,15,16,17,18,19,20].

Because EVs transfer a complex content of biological information (nucleic acids, lipids, and proteins), the manipulation of EV cargos represents a potential tool for drug and selective biomolecule delivery to target cells [21,22]. MicroRNAs (miRNAs) are a type of small noncoding RNA (sncRNA) which is able to modify the stability of messenger RNAs (mRNAs), usually silencing the gene expression in various cellular processes as cell apoptosis, angiogenesis, and inflammation [23]. Recent works have reported the presence of miRNAs in EVs released from human bronchial epithelial (HBE) cells of patients with asthma [24], and with chronic obstructive pulmonary disease (COPD) [25]. However, very few studies have analyzed the RNA cargo of EVs generated after ETS. Recently, Stassen and his group showed that tissue factor procoagulants are released from bronchial epithelial cells in response to contact with cigarette smoke extract through an EVs mechanism, suggesting a possible function of EVs after smoke exposure [26,27]. 

In this study, we analyzed the RNA composition of EVs derived from control and CSC-treated human small airway epithelial (SAE) cells using next generation sequencing (NGS). We observed that CSC exposure led to diverse signatures of new RNA molecules in EVs, especially snRNA types, such as miRNAs. We identified the targets of a novel miRNA, hsa-miR-3913-5p, significantly enriched in EVs derived in response to CSC treatment. Analysis of hsa-miR-3913-5p potential target genes and their biological functions suggest that this miRNA plays a role in lipid transport/binding and the regulation of gene transcription. Our data add novel information about the cargo of EVs released from AECs in response to ETS, which could be used to develop biomarkers for the diagnosis of cigarette smoke-related diseases, as well as for the development of future therapeutic approaches. 

## 2. Materials and Methods

### 2.1. Small Airway Epithelial Cultures and Stimulation with Cigarette Smoke Condensate

SAE cells (Lonza Inc., San Diego, CA, USA), derived from the terminal bronchioli of cadaveric donors, were grown in culture medium containing 7.5 mg/mL bovine pituitary extract (BPE), 0.5 mg/mL hydrocortisone, 0.5 µg/mL hEGF, 0.5 mg/mL epinephrine, 10 mg/mL transferrin, 5 mg/mL insulin, 0.1 µg/mL retinoic acid, 0.5 µg/mL triiodothyronine, 50 mg/mL gentamicin, and 50 mg/mL bovine serum albumin. CSC was prepared by smoking University of Kentucky’s standard research cigarettes on an Federal Trade Commission smoke machine, as previously described [28]. The total particulate matter collected was extracted with dimethyl sulfoxide (DMSO) to generate a 4% solution. Cell monolayers were plated in basal media (no supplemented added) for 4–6 h prior to CSC (1 μg/mL) or DMSO vehicle (control) exposure for 48 h.

### 2.2. Extracellular Vesicles Purification

**Figure 1:**
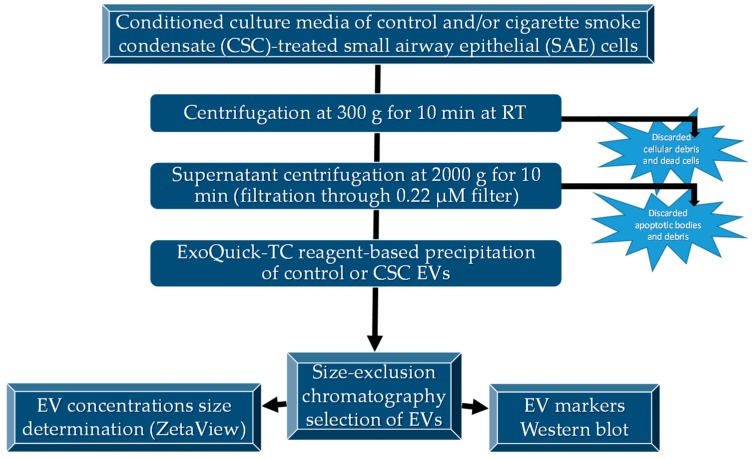
Diagram of EV isolation and characterization. EVs were isolated as described and characterized for size by ZetaView^®^ analysis and specific markers by Western blot analysis.

After CSC exposure, 100 mL of cell supernatant was centrifuged at 3000 g for 15 min at 4 °C for debris removal. The clear media was subjected to further cleaning by filtration through 0.22 μm sterile filter (Millipore, MA, USA) to remove any remaining debris. The filtered media was transferred to Amicon^®^ Ultra-15 centrifugation filters (Millipore, Billerica, MA, USA) and centrifuged at 2500 g for 35 min. ExoQuick-TC™ (System Biosciences, USA) reagent was added to the media, mixed thoroughly, and incubated overnight at 4 °C to precipitate the EVs. The following morning, the mixture was subjected to centrifugation at 1500 g for 30 min, and the EV pellet was washed and resuspended in filtered PBS. The resuspended EVs were passed through the Exo-spin^™^ columns (Cell Guidance Systems, St. Louis, MO, USA), and 300 μL of purified EVs were eluted from the column and used for experimental procedures. Protein concentration was determined using a protein assay kit from Bio-Rad, USA. The purified EVs were further characterized using nanoparticle size tracking and the determination of protein markers by Western blot analysis (Figure 1).

### 2.3. Nanoparticle Tracking Analysis with ZetaView^®^

EVs size distribution and number of particles were analyzed using the ZetaView PMX 110 (Particle Metrix GmbH, Meerbusch, Germany) and its corresponding software (Zeta-View^®^ 8.04.02, Particle Metrix GmbH). Samples of control or CSC EVs solution were run according to the manufacturer’s instructions and measured three times to ensure reproducibility. The machine was cleaned between samples using filtered water.

### 2.4. Western Blot Analysis for EVs Markers

EVs samples were lysed in a buffer (50mM TrisNaCl, 0.5% Triton, 300 mM NaCl) supplemented with protease and phosphatase inhibitor cocktail. Equal amount of proteins, 15 µg in total, were processed as described previously [20]. The primary antibodies for Western blot were rabbit anti-human CD63 (1:1000; System Biosciences), mouse anti-human Alix (1:800; Santa Cruz), mouse anti-human EpCAM (1:500; Santa Cruz), mouse anti-human Flotillin-1 (1:500; Santa Cruz), and mouse anti-human GM130 cis –Golgi (1:800; Santa Cruz). A densitometric analysis of band intensities was calculated using the UVP VisionWorks^®^ Life Science Software 8.0 RC 1.2 (UVP, Upland, CA, USA), verifying for nonsaturation and subtracting background.

### 2.5. Extraction of EVs RNA and Next Generation Sequencing (NGS)

RNA was extracted from control or CSC EVs by the phenol/chloroform method using all RNA-grade reagents and according to our published protocol [20]. Small RNA libraries were made using the QIAseq^®^ miRNA Library Kit (QIAGEN) following the manufacturer’s protocol. After Agilent Bioanalyzer analysis, the sample libraries were pooled and sequenced by the UTMB Next Generation Sequencing Core on an Illumina NextSeq550 (single end 75 base) using TruSeq SBS kit v3 (Illumina) and protocols defined by the manufacturer. The miRDeep2 software package, version 2.0.0.8, was used to trim adapter sequences from the reads and quantify miRNA read counts using the miRBase database, release 22. piRNAs were counted by mapping the trimmed reads to piRNA sequences downloaded from the DASHR database [29] using Bowtie version 1.2.2 with parameters -v2 -l18 -a -M10 -best -strata. For other small RNAs and protein coding genes, the reads were mapped to the hg38 reference with the same Bowtie parameters. Reads per gene were counted using the feature counts function of the subread programs [30] and the GENCODE release 29 annotation file. 

### 2.6. Reverse Transcription (RT)-PCR

To validate the up- and down- regulated miRNA and piRNA expressions, 1 microgram of isolated RNA was converted into cDNA using miScript II for miRNAs or the miScript Plant cDNA synthesis reagents (QIAGEN) for piRNAs species, following the manufacturer’s instructions. Since piRNAs have 2′-O-Me modifications on the 3′ terminal base and are refractory to polyadenylation, a ligation reaction was performed to overcome the polyadenylation step. Reverse transcription was performed following ligation. First, 1 µl cDNA served as a template for the PCR analysis using the miScript SYBR green PCR kit (QIAGEN) and custom miRNAs and piRNAs primers (QIAGEN). 

### 2.7. Predicted Targets of Hsa-miR-3913-5p and Functional Analysis

TargetScanHuman version 7.2, with a *context* ++ score, was used to predict the biological targets of hsa-miR-3913-5p identified in EVs; 19475 unique genes, 28353 transcripts, were scanned. Candidate target genes for hsa-miR-3913-5p were called whenever the gene was paired with the miRNA or its variant in the default predictions table of TargetScan. We used Panther interface to the Gene Ontology (GO) database to identify the most prevalent functions of the significant predicted target genes for hsa-miR-3913-5p. We chose three GO categories: biological process, cellular component, and molecular function. Relative frequencies were calculated against the entire human genome as background. *p* value < 0.05 was designated to be statistically significant. 

### 2.8. Statistical Analysis

EV size and concentration (*n* = 3), western blot (*n* = 4), NGS (*n* = 3), and RT-PCR (*n* = 4) are representative of independent experiments. The raw read counts of NGS analysis were normalized across all samples and then used for pairwise differential expression analysis using the R package DeSeq. Significant differentially-expressed RNAs were determined by *p* value with a threshold of 0.05. Log2 fold changes between samples were hierarchically clustered using Pearson correlation. Some miRNAs and piRNAs exhibited a large fold change on average, but the variance among samples was too high to call the difference significant. The fold change of RT-PCR experiments was calculated by the 2-ΔΔCT method and represented mean ± SEM using GraphPad Prism v4 (GraphPad Software). A *p* value < 0.05 was considered statistically significant using the student t-test statistics. 

## 3. Results

### 3.1. Characteristics of EVs Released by SAE Cells under Control and CSC Conditions

**Figure 2:**
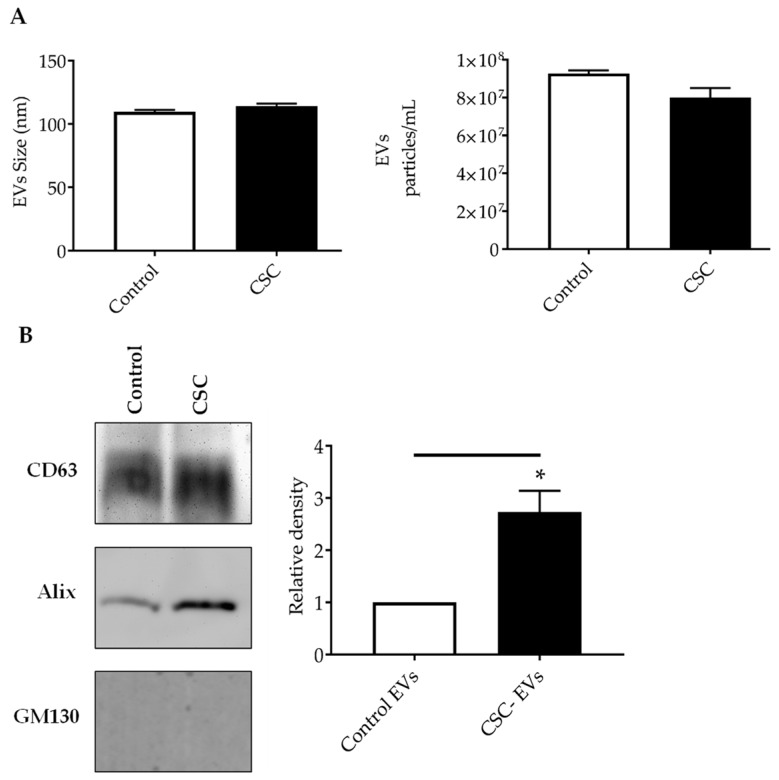
Characterization of EVs released from SAE cells under standard (control) or CSC treatment. (**A**) Absolute size determination (left panel) and concentration (right panel) of control and CSC- EVs by ZetaView^®^ PMX-110 analysis. The particles were tracked and sized based on Brownian motion. The absolute count of EVs was determined and expressed as particles/mL. (**B**) Western blot analysis of equal amounts of purified EVs (25μg) for CD63, Alix and GM130. Graph shows densitometric analysis of Alix in EVs as mean ± SEM. * indicates a statistical difference comparing CSC EVs versus control-EVs (*p* value < 0.05). EVs size and concentration, and western blot analysis are representative of three and four experiments, respectively.

We first investigated the generation and release of EVs from control and CSC-treated SAE cells. EVs were obtained using a two-step enrichment procedure: the first step was a reagent-specific precipitation, followed by size-exclusion chromatography, as described previously with modifications [20]. Following enrichment, EVs were characterized using two methods: particle sizing (size and concentration) and Western blot for the EV markers. We found that the average size of EVs from the control and CSC-treated cells were 109.5 nm and 114 nm, respectively. SAE cells of the control group produced an average of 9.2 × 10^7^ particles/mL, and SAE cells in the CSC-treated group produced an average of 8.0 × 10^7^ particles/mL (Figure 2A), indicating a similar particle release between control and treated cells. We confirmed the presence of EV markers, such as the tetraspanin CD63, and the programmed cell death 6 interacting protein Alix by Western blot analysis. The cis-Golgi matrix protein GM130, a control marker to monitor cellular contaminations in the preparations, was not detected as expected (Figure 2B). 

Of interest, there was a significant increase in Alix levels in CSC versus control EVs, while CD63 protein levels did not change for both conditions. EpCAM and Flotillin-1, other EV markers, were not detected in our samples (data not shown). 

### 3.2. EVs from Control and CSC-Treated SAE Cells Contain Small RNAs

We next determined the small RNA composition in EVs isolated from untreated or CSC-exposed SAE cells using NGS. The total reads for EVs isolated from control and CSC-treated cells were 35561353 and 3403131789, respectively. Unannotated represent the sequences that did not map to any known region of the human genome. We identified a broad range of RNA types in the EVs of both groups, such as ribosomal RNA (rRNA), protein coding, processed transcript and small noncoding RNAs (snRNAs), including transfer RNAs (tRNAs) small nucleolar RNAs (snoRNAs), microRNAs (miRNAs), Piwi-interacting RNA (piRNA), and miscellaneous RNAs (miscRNAs), as shown in Table 1. The percentage of RNA types in EVs was obtained by dividing the RNA type specific reads by total number of mapped reads. 

CSC treatment resulted in changes in the EV content of tRNAs, miRNAs, and miscRNAs, although only miRNA reached statistical significant difference, while levels of rRNAs, protein coding transcripts, and piRNAs did not change. Among the RNAs showing a difference in EV-derived from CSC-treated cells, tRNAs were the largest component of identified RNA species in EVs from control (9.1%) and CSC-treated cells (12.5%), followed by miRNAs (5.3% in EVs control and 7.7% in CSC EVs), miscRNAs (2.2% in EVs control and 3.52% in CSC EVs).

### 3.3. CSC Induces Changes in miRNAs Expression Profile in EVs from CSC-Treated SAE Cells

miRNAs are a diverse class of snRNA, about 22 nucleotides long, and are able to regulate gene expression [31,32]. Looking at their expression profiles, a total of 289 miRNAs were identified in EVs derived from control and CSC-treated cells (Table S1). Eight miRNAs demonstrated significant changes (*p* value < 0.05) in EVs derived from CSC-treated cells compared to control. The top upregulated (log2 fold change > 5) and downregulated (log2 fold change < 1) miRNAs are listed in Table 2. 

**Figure 3:**
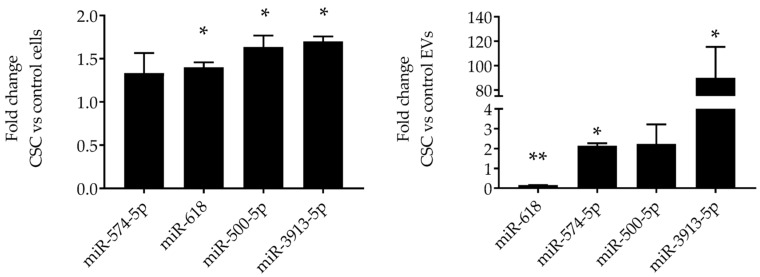
Validation of miRNAs expression in SAE cells and EVs. RNA extracted from SAE cells control and CSC-treated (left panel) and from control and CSC EVs (right panel) was subjected to miRNAs analysis by RT-PCR. Fold changes in miRNA expression were determined by 2-ΔΔCT method and represent mean ± SEM normalized to small nucleolar RNA C/D box 61 (SNORD61). * and ** indicates a statistical difference comparing CSC EVs versus control-EVs (* *p* value < 0.05; ** *p* value < 0.01). Data is representative of four independent experiments.

Out of those eight miRNAs, we confirmed the expression of three upregulated miRNAs (miR-3913-5p, miR-574-5p, miR-500a-5p) and one downregulated miRNA (miR-618) by RT-PCR in both control and CSC-exposed cells, as well as in EV derived from both conditions. Levels of miR-3913-5p, miR-574-5p, miR-500a-5p, and miR-618 were similar in CSC-treated cells compared to control cells (Figure 3, left panel), while miR-3913-5p, miR-574-5p, and miR-500a-5p levels were higher and miR-618 lower in EVs derived from CSC-treated cells compared to control (Figure 3, right panel), confirming the results obtained from NGS. miR-3913-5p was the top upregulated miRNA in CSC EVs, with the highest fold change. These results indicate an enrichment of these miRNAs in EVs compared to levels present in SAE cells.

piRNAs are a class of snRNAs (24–35 nucleotide long) also involved in epigenetic and post-transcriptional gene silencing mechanisms [33]. A total of 62 piRNAs were detected in our EV preparations (Table S2); we found that five were significantly downregulated and two upregulated in EVs isolated from CSC-treated cells, compared to control. The top downregulated piRNAs (<1.5 log2 fold change) and the top piRNAs upregulated (>5 fold change) are shown in Table 3. 

**Figure 4:**
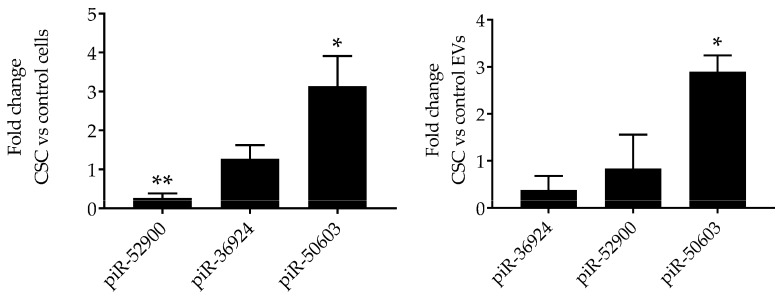
Validation of piRNAs expression in SAE cells and EVs. RNA extracted from control and CSC-treated (left panel) SAE cells and from control and CSC EVs (right panel) was subjected to piRNAs analysis by RT-PCR. Fold changes in piRNA expression were determined by 2-ΔΔCT method and represent mean ± SEM normalized to small nucleolar RNA C/D box 61 (SNORD61). * and ** indicates a statistical difference comparing CSC EVs versus control-EVs (* *p* value < 0.05; ** *p* value < 0.01). Data is representative of four independent experiments.

To validate the NGS results, we selected two downregulated piRNAs, i.e., piR-52900 and piR-36924, and the upregulated piR-50603, and confirmed their level of expression by RT-PCR in EVs control and CSC EVs, as well as in control and CSC-treated cells. We found that piR-50603 was upregulated in CSC-treated cells with a similar profile of expression in EVs derived from CSC-treated cells. The piR-52900 level was lower both in CSC-treated cells and CSC EV, while the piR-36924 level was lower only in CSC EVs but not in cells, compared to control, as shown in Figure 4. 

### 3.4. Potential Targets and Gene Ontology Prediction of the Upregulated Hsa-miR-3913-5p in CSC EVs 

Since hsa-miR-3913-5p was significantly enriched in EVs derived from CSC-treated SAE cells, we next characterized the predicted targets of hsa-miR-3913-5p using the human targets database of the TargetScan software package, v. 7.2 (TargetScan is being developed at the Whitehead Institute for Biomedical Research, Massachusetts Institute of Technology, Cambridge, MA, USA) [34,35]. We identified 5297 potential targets for the selected miRNA. Then, we selected 31 targets with a cumulative weighted *context* ++ score < −0.5, shown in Table 4. 

The Pleckstrin homology domain containing S1 (PLEKHS1) was the potential target with the highest score (−1.39). PLEKHS1 protein is highly expressed in bronchial epithelial cells and plays a role in intracellular signaling or as constituents of the cytoskeleton. Other high-scoring targets included the germinal center-associated signaling and motility protein (GCSAM) and B-cell linker protein (BLNK). GCSAM has been involved in the negative regulation of lymphocyte motility [36]. BLNK, highly expressed in the mouth mucosa, functions as central linker protein for the B cell receptor and the activation of nuclear factor kappa-B [37,38]. 

We next used the Gene Ontology (GO) functional classification tool [39,40] in order to determine the cell component, biological processes, and molecular function of the top predict targets of hsa-miR-3913-5p (Table 5). The most enriched biological process included lipid transport and the regulation of mRNA stability, with lipid binding being the molecular function group with the highest enrichment. 

## 4. Discussion

The aim of this study was to characterize snRNA content using high-throughput technologies in EVs derived from normal AECs after exposure to CSC, as a proxy for ETS [28,41], since the role of EVs and exosomes in the pathogenesis of cigarette smoke-related diseases is still mostly unknown. A recent in vitro study reported that HBE cells exposed to cigarette smoke extract (CSE) released EVs, and EVs from both normal and CSE-treated cell conditions displayed the expression of CD63, the EV marker. Also, the size and number of EVs derived from control and CSE-treated HBE cells did not vary significantly between the two groups [25]. The presence of the CD63 marker was also confirmed in EVs isolated from untreated and CSE-treated immortalized HBE cells (BEAS 2B), and while no significant differences of EVs size were detected in both conditions, CSE exposure increased the amount of EVs released from BEAS 2B cells [42]. Similar to the published finding of Xu’s team, our data suggested that human SAE cells are able to secrete EVs under control and cigarette smoke exposure conditions in an in vitro model. Although the differential centrifugation technique is widely used for the EV purification, we opted for the two-step procedure, i.e., precipitation reagent solution and size exclusion chromatography (SEC), as it is associated with a higher purity of EVs than differential centrifugation alone [43]. We found that CSC exposure did not cause significant differences in the concentration and size of SAE-derived EVs. CD63 and Alix, EVs markers, were present in both groups, with EVs isolated from CSC-SAE cells showing increased Alix content. A similar result was reported for the EV isolated from CSC-exposed human monocytes [44]. 

Our RNA sequencing analysis revealed that EVs isolated from CSC-treated SAE cells displayed a different amount of various small noncoding RNAs (sncRNA) compared to the control EVs. We decided to explore two snRNA classes associated with the gene regulation processes: miRNAs and piRNAs. miRNAs are 22 nucleotides long. miRNAs usually functions in gene silencing via translational repression or target degradation [45]. NGS analysis showed that five miRNAs were upregulated (e.g., miR-3913-5p, miR-574-5p, miR-656-5p, and miR-3180-5p), and three downregulated (miR-618, miR-222-5p, and miR-130b-5p) in EVs derived from CSC-exposed cells, compared to control cells. Real-time PCR confirmed that CSC treatment led to the upregulation and enrichment of novel miRNAs in EVs, namely miR-3913-5p, miR-574-5p, and miR-500a-5p, whose function is still unexplored. Of the downregulated miRNAs, it has been previously reported that miR-222 was downregulated in the lungs of rats exposed to cigarette smoke [46]. Changes in small RNA (including miRNA) composition have been reported in EVs derived from HBE cells after CSE treatment. Recent studies demonstrated that two miRNAs, miR-210 and miR-21, were upregulated in EVs after cell exposure to CSE [25,47]. When using BEAS 2B cells, He and colleagues found that a low percentage of CSE reduced the amount of miR-21 while a higher CSE percentage increased the miR-21 content of EVs [48]. In our experiments, miR-21 was detectable in both CSC and control EVs, with a marginal increase in the CSC ones, which, however, was not statistically significant. The different type of airway epithelia cells and cigarette smoke sources could be responsible for the differences in our results. For example, Baskoro demonstrated that SAE cells exhibit a different proinflammatory response to cigarette smoke exposure compared to HBEs [49]. 

Since hsa-miR-3913-5p was the highest upregulated miRNA in CSC EVs, we focused on the identification of potential genes regulated by this miRNA using the TargetScan software, and classified them into functional groups. PLEKHS1 was the potential target with the highest score, and lipid transport, lipid binding, and regulation of mRNA stability were the most enriched biological and molecular function groups. Lipids are critical components of airway mucosa, and cigarette smoke exposure has been shown to alter lipid homeostasis in mouse alveolar macrophages [50]. It is known that PLEKH domains have a specific affinity for binding lipids product as phospholipid molecules [51]. APOA4, NME4, and S100-A10 protein (S100A10) represented the most significant groups of targets of hsa-miR-3913-5p involved in lipid binding (*p* <0.05). Although ApoA4 has not been investigated in the context of smoke exposure, ApoA-I, another apopoliprotein, exhibits multiple protective features in normal and pathological lung conditions (asthma, emphysema, influenza, and lung cancer). In particular, ApoA-I displayed anti-inflammatory and antioxidant properties in a mouse model of cigarette smoke-induced emphysema [52]. Future studies will focus on identifying the actual targets regulated by hsa-miR-3913-5p and their biological function.

In conclusion, a better understanding of EV generation and composition in response to ETS exposure, as well as the identification of the biological targets of their cargo, could lead to their potential utilization as diagnostic biomarkers, and to the identification of novel therapeutic targets of smoke and ETS-dependent diseases. 

## Figures and Tables

**Table 1:** Percentage of RNA types in EVs from control and CSC-treated SAE cells. Data represent the mean ± SEM of three independent experiments.

RNA Type	% Control EVs	% CSC EVs	*p* Value
protein coding	2.9 ± 1.3	3 ± 1.4	0.4
processed transcript	1.5 ± 0.7	1.3 ± 0.7	0.4
miRNA	5.0 ± 0.7	7.5 ± 0.8	0.04
tRNA	9.1 ± 4.0	12.5 ± 6.2	0.3
piRNA	0.4 ± 0.01	0.38 ± 0.02	0.3
snoRNA	6.1± 3.0	5.3 ± 2.9	0.4
rRNA	8.1 ± 0.3	8.4 ± 0.9	0.4
miscRNA	1.4 ± 0.6	2.3 ± 1.0	0.2
snRNA	0.8 ± 0.4	0.9 ± 0.4	0.4
unannotated	63.6 ± 9.3	57 ± 11.3	0.3

**Table 2:** Top five miRNAs upregulated and three miRNAs downregulated in CSC EVs. Data represent the average of three independent experiments.

miRNAs	Log2 Fold Change	*p* Value	Read Count (Avg)	
			CSC EVs	Control EVs
hsa-miR-3913-5p	10.28	0.0003	356.00	1.62
hsa-miR-574-5p	10.25	0.0006	183.57	0
hsa-miR-656-5p	9.76	0.0014	126.40	0
hsa-miR-3180-5p	9.21	0.0022	39.98	0
hsa-miR-500a-5p	8.91	0.0033	84.5	0
hsa-miR-618	−9.25	0.00241	0	76.053
hsa-miR-222-5p	−8.78	0.00472	0	55.44
hsa-miR-130b-5p	−8.20	0.005	0	38.35

**Table 3:** Top five piRNAs downregulated and two piRNAs upregulated in CSC EVs. Data represent the average of three independent experiments.

piRNAs	Log2 Fold Change	*p* Value	Read Count (Avg)	
			CSC EVs	Control EVs
piR-36705	−10.19	0.002	0	191.02
piR-37183	−10.19	0.002	0	191.02
piR-59260	−10.19	0.002	0	191.02
piR-36924	−10.24	0.002	0.32	358.4
piR-52900	−10.28	0.004	0	211.63
piR-31985	10.52	0.004	498.48	0
piR-50603	9.78	0.001	276.51	0

**Table 4:** Predicted target genes of hsa-miR-3913-5p.

Target Gene	Cumulative Weighted *Context* ++ Score	Target Gene	Cumulative Weighted *Context* ++ Score
PLEKHS1	−1.39	AC005477.1	−0.58
GCSAM	−1	FCGR2A	−0.58
BLNK	−0.75	ETNK1	−0.55
ACMSD	−0.74	MYF5	−0.55
AP000708.1	−0.72	NME4	−0.54
KCNAB2	−0.7	APOA4	−0.54
IQCK	−0.7	KRTAP4-6	−0.53
RHAG	−0.65	AL049747.1	−0.53
S100A10	−0.65	ZNF740	−0.53
NHLH1	−0.61	SEPT3	−0.53
OR51F2	−0.61	ITGAE	−0.53
VSNL1	−0.6	PRKCDBP	−0.52
ERGIC1	−0.6	TPMT	−0.52
PYY	−0.59	C21ORF33	−0.5
YWHAZ	−0.59	RP11-321F6.1	−0.5
PSMC4	−0.59		

**Table 5:** GO term enrichment functional analysis. Top gene targets categorized in three biologically-relevant categories.

Term	Genes	Enrichment
**Biological process**		
lipid transport	NME4, APOA4	19.21
regulation of mRNA stability	YWHAZ, PSMC4	14.17
extracellular matrix organization	ITGAE, MYF5	7.44
RNA polymerase II promoter	MYF5, NHLH1	2.84
**Cellular component**		
cytosol	APOA4, YWHAZ, KCNAB2, PSMC4, VSNL1, ACMSD, ETNK1, TPMT, BLNK	2.06
membrane	KCNAB2, PSMC4, VSNL1, ETNK1, RHAG, ERGIC1	2.07
blood microparticle	APOA4, YWHAZ	9.99
plasma membrane	KCNAB2, ITGAE, OR51F2, ETNK1, GCSAM, FCGR2A, RHAG, BLNK	1.47
extracellular exosome	APOA4, YWHAZ, ACMSD, S100A10, FCGR2A, TPMT	1.62
cytoskeleton	SEPT3, KCNAB2	4.09
cell junction	SEPT3, KCNAB2	3.3
integral component of membrane	KCNAB2, ACMSD, OR51F2, ETNK1, S100A10, FCGR2A, RHAG, ERGIC1	1.17
**Molecular function**		
lipid binding	NME4, APOA4, S100A10	13.97
protein binding	YWHAZ, SEPT3, MYF5, ACMSD, GCSAM, S100A10, PRKCDBP, ERGIC1, APOA4, NME4, PSMC4, VSNL1, ETNK1, FCGR2A, PYY, PLEKHS1, BLNK	1.36
transcriptional activation only	MYF5, NHLH1	14.80
hydrolase activity	PSMC4, ACMSD	7.36
protein kinase binding	YWHAZ, GCSAM	3.74

## Supplementary Materials

The following are available online at https://www.mdpi.com/2073-4409/8/12/1652/s1, Table S1: Complete list of miRNAs expressed in EVs control and upon CSC exposure, Table S2: Complete list of piRNAs expressed in EVs control and upon CSC exposure.
