# Supplementary material for: Cigarette Smoke Condensate Exposure Changes RNA Content of Extracellular Vesicles Released from Small Airway Epithelial Cells
Source: Cells. 2019 Dec 17;8(12):1652. doi: 10.3390/cells8121652 (PMC6953119; doi:10.3390/cells8121652)
Supplement: Supplementary file 1 [file cells-08-01652-s001.pdf]

Supplementary Table 1. Complete list of miRNAs expressed in EVs control and upon CSC exposure**.**

miRNAs	Log2 Fold Change	*p* value	lfcSE	Read Count (Avg)	
				CSC EVs	Control EVs
1. hsa-miR-23b-5p	-21.60	7.55E-09	3.738545311	0	40.56
2. hsa-miR-4750-5p	21.33	4.89E-08	3.909279422	21.32	0
3. hsa-miR-3913-5p	10.28	0.00034	2.86842392	356.00	1.62
4. hsa-miR-574-5p	10.25	0.00065	3.006666452	183.57	0
5. hsa-miR-656-5p	9.76	0.00148	3.070950116	126.40	0
6. hsa-miR-3180-5p	9.22	0.00221	3.012329946	39.98	0
7. hsa-miR-618	-9.25	0.00241	3.048894118	0	76.053
8. hsa-miR-500a-5p	8.91	0.00330	3.033889994	84.5	0
9. hsa-miR-222-5p	-8.79	0.00473	3.110620434	0	55.44
10. hsa-miR-130b-5p	-8.20	0.00594	2.982047994	0	38.35
11. hsa-miR-24-1-5p	-8.61	0.00614	3.141108097	0	49.06
12. hsa-miR-3943	-8.31	0.00942	3.20260571	0	40.39
13. hsa-miR-516a-5p	7.61	0.02092	3.295435878	34.00	0
14. hsa-miR-548ab	-7.76	0.02149	3.374890665	0	27.38
15. hsa-miR-1252-5p	-7.85	0.02196	3.426160125	0	28.51
16. hsa-miR-4326	-7.55	0.03339	3.550194415	0	23.19
17. hsa-miR-153-5p	-7.49	0.03908	3.628264038	0	22.13
18. hsa-miR-25-5p	-7.15	0.05795	3.77265891	0	17.56
19. hsa-miR-6513-5p	-6.71	0.08647	3.913571587	0	12.92
20. hsa-miR-6875-5p	-6.69	0.08735	3.913659248	0	14.60
21. hsa-miR-1275	6.64	0.08962	3.910512095	14.31	0
22. hsa-miR-337-5p	-6.42	0.10127	3.915103872	0	11.18
23. hsa-miR-518e-5p	-6.36	0.10453	3.915457008	0	10.72
24. hsa-miR-519a-5p	-6.36	0.10453	3.915457008	0	10.72
25. hsa-miR-519b-5p	-6.36	0.10453	3.915457008	0	10.72
26. hsa-miR-519c-5p	-6.36	0.10453	3.915457008	0	10.72

27.	hsa-miR-522-5p	-6.36	0.10453	3.915457008	0	10.72
28.	hsa-miR-523-5p	-6.36	0.10453	3.915457008	0	10.72
29.	hsa-miR-27a-5p	6.24	0.11088	3.911712784	14.06	0
30.	hsa-miR-10398-3p	-6.23	0.11177	3.916261248	0	9.8
31.	hsa-miR-1273h-5p	-6.21	0.11282	3.916381035	0	9.12
32.	hsa-miR-302a-5p	-6.19	0.11375	3.916486121	0	9.58
33.	hsa-miR-34b-5p	-6.16	0.11580	3.916721853	0	9.35
34.	hsa-miR-6733-5p	6.04	0.12249	3.912423386	12.37	0
35.	hsa-miR-516b-5p	5.95	0.12866	3.91281755	11.56	0
36.	hsa-miR-29b-1-5p	5.88	0.13317	3.913112319	11.02	0
37.	hsa-miR-4665-5p	-5.87	0.13413	3.918924846	0	7.2
38.	hsa-miR-19b-1-5p	-5.80	0.13869	3.919501408	0	7.3
39.	hsa-miR-296-5p	-5.76	0.14151	3.919864761	0	6.69
40.	hsa-miR-1911-5p	5.73	0.14330	3.913797283	9.95	0
41.	hsa-miR-329-5p	-5.71	0.14519	3.9203437	0	6.84
42.	hsa-miR-548ar-3p	5.69	0.14610	3.913992263	9.68	0
43.	hsa-miR-505-5p	5.59	0.15229	3.907945181	17.48	0.2
44.	hsa-miR-23a-5p	5.48	0.16167	3.915119435	6.4	0
45.	hsa-miR-2276-5p	5.43	0.16584	3.915434505	8.07	0
46.	hsa-miR-10400-5p	4.13	0.16657	2.98372848	39.93	2.12
47.	hsa-miR-767-5p	5.38	0.16972	3.915732765	7.8	0
48.	hsa-miR-939-5p	5.38	0.16972	3.915732765	7.8	0
49.	hsa-miR-4705	5.22	0.18269	3.916764853	6.99	0
50.	hsa-miR-624-5p	5.22	0.18269	3.916764853	6.99	0
51.	hsa-miR-128-1-5p	-5.20	0.18538	3.926095691	0	4.7
52.	hsa-miR-1271-5p	-4.04	0.18891	3.075805963	1.5	27.15
53.	hsa-miR-215-5p	3.20	0.19098	2.449946764	26.46	2.81
54.	hsa-miR-4804-5p	4.98	0.20395	3.918577503	5.9	0
55.	hsa-miR-365b-5p	4.91	0.21018	3.91913822	5.64	0
56.	hsa-miR-4429	4.81	0.21863	3.90964474	10.26	0.54

57. hsa-miR-3180	4.77	0.22406	3.920436114	5.1	0
58. hsa-miR-3180-3p	4.77	0.22406	3.920436114	5.1	0
59. hsa-miR-378a-5p	4.77	0.22406	3.920436114	5.1	0
60. hsa-miR-1283	4.69	0.23183	3.921192821	4.84	0
61. hsa-miR-4999-5p	4.69	0.23183	3.921192821	4.84	0
62. hsa-miR-433-5p	4.61	0.24024	3.922038216	4.57	0
63. hsa-miR-29b-2-5p	3.36	0.24507	2.88738769	98.48	10.27
64. hsa-miR-589-5p	-2.75	0.24924	2.38676978	57.03	410.17
65. hsa-miR-1976	4.47	0.25425	3.923503976	3.21	0
66. hsa-miR-4700-5p	4.47	0.25425	3.923503976	3.21	0
67. hsa-miR-193a-5p	2.90	0.25540	2.551593768	41.84	6.39
68. hsa-miR-137-5p	4.43	0.25943	3.924065689	4.03	0
69. hsa-miR-182-5p	2.52	0.26450	2.262667467	945.23	211.37
70. hsa-miR-378g	-3.76	0.26709	3.383773086	0.77	11.18
71. hsa-miR-6511a-5p	4.29	0.27342	3.912577224	7.26	0.30
72. hsa-miR-6511b-5p	4.29	0.27342	3.912577224	7.26	0.30
73. hsa-miR-299-5p	3.21	0.27654	2.949056407	42.28	4.79
74. hsa-miR-2277-5p	3.20	0.28152	2.968535031	36.87	4.10
75. hsa-miR-122-5p	-2.51	0.28496	2.346184422	108.00	702.65
76. hsa-miR-376b-5p	4.10	0.29625	3.928367274	3.22	0
77. hsa-miR-376c-5p	4.10	0.29625	3.928367274	3.22	0
78. hsa-miR-92a-1-5p	4.10	0.29625	3.928367274	3.22	0
79. hsa-miR-200a-5p	3.15	0.29749	3.021761713	3	4.3
80. hsa-miR-22-5p	-2.26	0.29750	2.173041149	181.63	946
81. hsa-miR-155-5p	2.14	0.30688	2.090974754	389.13	98.36
82. hsa-miR-342-5p	-3.44	0.31073	3.39047069	1.88	18.74
83. hsa-miR-196b-5p	2.58	0.32449	2.62068812	113.10	18.70
84. hsa-miR-885-5p	-2.11	0.33350	2.184689323	145.36	685.27
85. hsa-miR-6841-5p	3.69	0.34873	3.935516423	2.42	0
86. hsa-miR-499a-5p	-2.66	0.35510	2.876642036	29.86	181.28

87. hsa-miR-15b-5p	1.91	0.38977	2.215884807	411.95	132.28
88. hsa-miR-4497	3.10	0.43221	3.949741725	1.61	0
89. hsa-miR-548b-5p	3.10	0.43221	3.949741725	1.61	0
90. hsa-miR-143-5p	-2.28	0.43448	2.91169751	29.59	133.32
91. hsa-miR-323a-5p	-2.27	0.43552	2.908853776	29.59	133.32
92. hsa-miR-33a-5p	1.95	0.44227	2.540022493	76.15	20.15
93. hsa-miR-2682-5p	-1.91	0.45004	2.526573093	57.29	211.49
94. hsa-miR-224-5p	1.85	0.45917	2.494286714	987.88	328.89
95. hsa-miR-3065-5p	-2.15	0.46389	2.92911213	34.70	142.45
96. hsa-miR-423-5p	1.29	0.46412	1.766551517	1549.51	781.72
97. hsa-miR-582-5p	-1.85	0.47086	2.564412041	110.05	381.12
98. hsa-miR-592	2.05	0.47396	2.862857673	136.89	34.23
99. hsa-miR-148a-5p	1.77	0.49318	2.585594412	66.09	19.77
100.hsa-miR-16-5p	1.12	0.51672	1.721735439	6294.44	3529.74
101.hsa-miR-425-5p	1.40	0.51987	2.177931952	1996.60	813.89
102.hsa-miR-199a-5p	-1.17	0.52372	1.839179128	139.81	324.08
103.hsa-miR-378j	-2.46	0.54128	4.030263851	0	0.68
104.hsa-miR-362-5p	1.76	0.54248	2.888875404	179.28	55.69
105.hsa-miR-487a-5p	1.54	0.54702	2.556624781	57.93	20.31
106.hsa-miR-1224-5p	1.60	0.54761	2.662515126	61.51	19.92
107.hsa-miR-1179	1.80	0.54966	3.008772166	33.36	10.04
108.hsa-miR-1296-5p	-1.00	0.55658	1.693924455	270.81	563.15
109.hsa-miR-744-5p	-0.96	0.56246	1.662016251	697.74	1496.44
110.hsa-miR-5701	1.75	0.56395	3.034137199	32.03	9.43
111.hsa-miR-577	1.65	0.56990	2.904364292	61.16	19.85
112.hsa-miR-493-5p	-1.07	0.57124	1.889258184	120.48	299.13
113.hsa-miR-339-5p	-1.58	0.58687	2.910939582	35.77	101.39
114.hsa-miR-10396b-3p	-1.97	0.58694	3.624276545	5.64	20.38
115.hsa-miR-374a-5p	-0.89	0.59003	1.643721658	764.49	3
116.hsa-miR-151a-5p	-0.91	0.59643	1.714393588	275.72	550.77
117.hsa-miR-151b	-0.91	0.59643	1.714393588	275.72	550.77
118.hsa-miR-301a-5p	1.55	0.59772	2.929003024	60.30	3
119.hsa-miR-584-5p	0.87	0.60367	1.681961902	264.96	165.28

120.hsa-miR-181d-5p	1.05	0.60604	2.026475756	2267.11	1185.17
121.hsa-miR-18a-5p	1.49	0.60871	2.909298189	63.01	23.51
122.hsa-miR-1468-5p	1.47	0.62461	3.006405347	35.21	15.97
123.hsa-miR-432-5p	0.87	0.62881	1.794088418	993.84	664.13
124.hsa-miR-770-5p	-1.45	0.63392	3.044593663	12.10	31.49
125.hsa-miR-192-5p	0.97	0.63472	2.039057801	2527.67	1408.34
126.hsa-miR-671-5p	0.85	0.63509	1.782086254	199.26	135.57
127.hsa-miR-374b-5p	-0.90	0.64077	1.922118611	741.16	1382.58
128.hsa-miR-383-5p	1.16	0.64700	2.540968602	336.53	151.15
129.hsa-miR-1306-5p	-1.19	0.65104	2.621171399	44.68	131.68
130.hsa-miR-431-5p	-1.43	0.66010	3.255263313	6.72	17.26
131.hsa-miR-142-5p	-1.02	0.66476	2.346610851	165.84	354.34
132.hsa-miR-485-5p	-1.10	0.66504	2.534274953	112.64	234.58
133.hsa-miR-708-5p	-0.71	0.66599	1.638495031	1488.84	2716.25
134.hsa-miR-106b-5p	0.93	0.66748	2.159945717	478.58	217.37
135.hsa-miR-20b-5p	-1.09	0.66756	2.538906438	112.64	234.58
136.hsa-miR-15a-5p	1.08	0.66828	2.521788405	106.74	50.74
137.hsa-miR-181c-5p	0.89	0.67956	2.153103742	654.28	3
138.hsa-miR-877-5p	0.93	0.68104	2.254184155	63.01	33.31
139.hsa-miR-345-5p	0.84	0.69240	2.114281554	654.28	3
140.hsa-miR-324-5p	-1.15	0.69461	2.930985298	64.29	137.85
141.hsa-miR-1250-5p	1.00	0.69986	2.597117583	52.69	26.01
142.hsa-miR-651-5p	-1.28	0.70106	3.334778854	7.53	16.88
143.hsa-miR-96-5p	1.01	0.70316	2.642762036	181.28	116.66
144.hsa-miR-103a-1-5p	1.53	0.70363	4.028676871	0.53	0
145.hsa-miR-149-5p	0.62	0.70559	1.6426674	3027.89	2238.43
146.hsa-miR-150-5p	-0.95	0.70781	2.521480275	72.44	139.67
147.hsa-miR-144-5p	-0.93	0.71171	2.524219268	47.32	91.97
148.hsa-miR-3064-5p	-1.48	0.71712	4.07681091	0	0.30
149.hsa-miR-548au-5p	-1.48	0.71712	4.07681091	0	0.30
150.hsa-miR-4488	0.74	0.71784	2.040281654	190.87	149.25
151.hsa-miR-127-5p	0.63	0.71949	1.752647122	696.70	493.66
152.hsa-let-7a-5p	0.61	0.71993	1.705086177	696.70	493.66
153.hsa-miR-125b-5p	0.58	0.72275	1.646037947	26606.13	20221
154.hsa-miR-4454	-1.44	0.72482	4.078371188	0	0.22

155.hsa-miR-518d-5p	-1.44	0.72482	4.078371188	0	0.22
156.hsa-miR-520c-5p	-1.44	0.72482	4.078371188	0	0.22
157.hsa-miR-526a-5p	-1.44	0.72482	4.078371188	0	0.22
158.hsa-miR-6805-5p	-1.44	0.72482	4.078371188	0	0.22
159.hsa-miR-346	0.86	0.73435	2.527851963	120.88	68.38
160.hsa-miR-135b-5p	0.89	0.73623	2.635601062	93.11	60.23
161.hsa-miR-196a-5p	0.99	0.73745	2.940669417	70.92	36.96
162.hsa-miR-154-5p	-0.85	0.73769	2.538518752	74.10	135.04
163.hsa-miR-1287-5p	-0.92	0.74148	2.779924635	275.72	550.77
164.hsa-miR-140-5p	0.71	0.74348	2.170692981	839.87	573.27
165.hsa-miR-219a-5p	-0.54	0.74398	1.654909939	329.75	574.14
166.hsa-miR-378c	-0.80	0.75229	2.527591579	104.35	179.15
167.hsa-miR-9985	0.64	0.75611	2.04752752	78.75	52.76
168.hsa-miR-10a-5p	0.68	0.75811	2.206754872	2197.03	1427.95
169.hsa-miR-212-5p	-0.78	0.75956	2.540527678	34.10	58.27
170.hsa-miR-125a-5p	0.51	0.75957	1.68235417	11247.08	9420.82
171.hsa-miR-625-5p	-1.14	0.76158	3.758762933	3.49	7.53
172.hsa-miR-211-5p	-1.01	0.76282	3.337657673	8.60	15.97
173.hsa-miR-17-5p	0.51	0.76325	1.705650178	698.72	568.76
174.hsa-miR-504-5p	0.65	0.76382	2.166626473	78.73	52.79
175.hsa-let-7d-5p	0.52	0.77096	1.779530385	698.72	568.76
176.hsa-miR-12136	-0.74	0.77394	2.561945362	30.17	50.97
177.hsa-miR-146a-5p	-0.46	0.77964	1.655213041	2458.761	3887.75
178.hsa-miR-30c-5p	-0.45	0.78462	1.647884222	2458.761	3887.75
179.hsa-miR-1246	0.74	0.78562	2.716376432	190.87	149.25
180.hsa-miR-548d-5p	-0.81	0.78731	2.987317324	104.35	179.15
181.hsa-miR-340-5p	-0.44	0.79101	1.669392222	2991.69	4260.29
182.hsa-miR-186-5p	0.44	0.79130	1.645032371	1946.51	1552.32
183.hsa-miR-361-5p	0.41	0.80142	1.64744133	882.08	736.27
184.hsa-let-7f-5p	0.40	0.80702	1.643033011	46571.28	40717.04
185.hsa-miR-629-5p	-0.68	0.80852	2.801031647	20.03	30.12
186.hsa-miR-500b-5p	0.94	0.81754	4.055364473	0.25	39.62
187.hsa-miR-34a-5p	0.42	0.81975	1.860173601	982.18	736.24
188.hsa-miR-34c-5p	0.39	0.81988	1.691383245	1578.54	1271.80

189.hsa-miR-105-5p	0.61	0.82220	2.716142931	50.87	33.40
190.hsa-miR-539-5p	-0.57	0.82230	2.540075515	92.94	133.11
191.hsa-miR-1843	0.66	0.82450	2.979521725	52.45	31.72
192.hsa-let-7e-5p	0.36	0.82873	1.644542526	4921.39	4457.17
193.hsa-miR-4510	0.56	0.82994	2.621062035	6.57	4.80
194.hsa-miR-204-5p	0.47	0.83082	2.189334891	1035.42	841.99
195.hsa-miR-93-5p	0.34	0.83540	1.658832465	1718.89	1547.53
196.hsa-miR-135a-5p	-0.51	0.84140	2.556320601	248.64	350.44
197.hsa-miR-450a-5p	0.53	0.84550	2.697291521	91.07	66.04
198.hsa-miR-134-5p	0.32	0.85062	1.690837426	683.61	641.49
199.hsa-miR-1248	0.47	0.85336	2.530125199	155.51	110.68
200.hsa-miR-129-5p	-0.30	0.85516	1.655261357	2509.62	3300.46
201.hsa-miR-455-5p	0.47	0.85692	2.587268113	597.57	431.70
202.hsa-miR-7-5p	0.30	0.85898	1.679850945	11977.5	95736.83
203.hsa-let-7g-5p	-0.29	0.85922	1.625156639	26184.18	34868.71
204.hsa-miR-1260a	-0.53	0.86753	3.184729213	6.12	8.36
205.hsa-miR-411-5p	-0.27	0.87252	1.683714434	2100.67	2653.82
206.hsa-miR-548ay-5p	-0.45	0.87656	2.878275403	11.92	16.81
207.hsa-miR-484	0.33	0.87801	2.134456352	301.20	258.26
208.hsa-miR-28-5p	0.36	0.87819	2.347161815	170.98	130.00
209.hsa-miR-30e-5p	0.25	0.88029	1.647080096	10516.81	9459.96
210.hsa-miR-30b-5p	-0.25	0.88137	1.651125779	3553.204	4455.659
211.hsa-miR-1285-5p	0.60	0.88223	4.064640391	0.26	29.28
212.hsa-miR-378b	0.60	0.88223	4.064640391	0.26	0
213.hsa-miR-6849-5p	0.60	0.88223	4.064640391	0.26	0
214.hsa-miR-654-5p	-0.55	0.88390	3.749991166	5.6	7.6
215.hsa-miR-382-5p	-0.24	0.88874	1.681250756	963.38	1295.05
216.hsa-miR-451a	-0.24	0.88964	1.737013872	9673.09	11689.78
217.hsa-miR-377-5p	0.37	0.89111	2.734050882	30.03	22.35
218.hsa-miR-146b-5p	0.22	0.89421	1.673009766	3969.57	3612.88
219.hsa-miR-32-5p	0.34	0.89544	2.563367491	373.05	296.91
220.hsa-miR-491-5p	0.30	0.89778	2.322501205	94.25	81.76
221.hsa-miR-139-5p	0.21	0.90182	1.693334416	8680.12	7994.139
222.hsa-miR-424-5p	-0.35	0.90602	2.960112975	41.96	50.50
223.hsa-miR-532-5p	-0.19	0.90700	1.654135447	500.52	636.92
224.hsa-miR-26b-5p	0.19	0.91034	1.64932747	10081.8	10293.23
225.hsa-miR-10b-5p	0.27	0.91110	2.455681396	1086.32	897.67
226.hsa-miR-181b-5p	-0.18	0.91181	1.664790223	6748.65	8091.37
227.hsa-miR-20a-5p	0.18	0.91275	1.634907538	1401.17	1351.05

228.hsa-miR-136-5p	-0.28	0.91386	2.551116401	233.41	277.79
229.hsa-miR-126-5p	-0.28	0.91497	2.598664414	502.79	594.81
230.hsa-miR-221-5p	0.17	0.91911	1.679095296	518.49	495.39
231.hsa-miR-370-5p	0.41	0.91981	4.070852393	0.126	0
232.hsa-miR-4508	0.41	0.91981	4.070852393	0.12	0
233.hsa-miR-548at-3p	0.41	0.91981	4.070852393	0.12	0
234.hsa-miR-628-5p	-0.24	0.92344	2.525681434	108.24	125.43
235.hsa-miR-374c-5p	-0.34	0.93233	4.062529912	0.26	0.22
236.hsa-miR-1277-5p	-0.31	0.93320	3.743989829	0.26	0.23
237.hsa-miR-181a-5p	-0.14	0.93333	1.625486657	27884.49	33357.72
238.hsa-miR-124-5p	-0.21	0.93513	2.549879992	10.74	12.93
239.hsa-miR-422a	-0.31	0.93526	3.860331296	1.04	1.29
240.hsa-miR-409-5p	-0.17	0.93579	2.171494093	436.56	519.64
241.hsa-miR-30a-5p	0.13	0.93660	1.644725512	21984.7	21399.35
242.hsa-miR-148b-5p	-0.23	0.93876	3.054639902	11.94	13.00
243.hsa-miR-138-5p	-0.12	0.94084	1.638220501	3243.67	3948.77
244.hsa-miR-769-5p	0.17	0.94164	2.270640447	436.56	519.64
245.hsa-miR-190a-5p	0.19	0.94256	2.576006745	133.81	120.63
246.hsa-miR-99a-5p	-0.11	0.94541	1.633407757	62200.19	72163.36
247.hsa-miR-490-5p	0.18	0.94701	2.725217941	132.73	121.78
248.hsa-miR-132-5p	-0.15	0.94748	2.220096763	1133.20	1289.34
249.hsa-miR-2110	-0.14	0.94875	2.251381047	113.94	97.15
250.hsa-miR-106a-5p	-0.12	0.94943	1.90608415	290.30	406.13
251.hsa-miR-380-5p	0.22	0.95005	3.485300431	11.02	8.74
252.hsa-miR-660-5p	0.11	0.95030	1.690285528	219.47	222.31
253.hsa-miR-379-5p	0.10	0.95321	1.74988574	2306.65	2343.01
254.hsa-miR-369-5p	-0.13	0.95520	2.282497097	96.39	107.87
255.hsa-miR-548ad-5p	-0.14	0.95868	2.7721208	19.69	22.52
256.hsa-miR-548ae-5p	-0.14	0.95868	2.7721208	19.69	22.52
257.hsa-miR-185-5p	0.08	0.95895	1.638687025	1018.09	1077.79
258.hsa-miR-330-5p	-0.08	0.96116	1.657961878	272.79	314.26
259.hsa-let-7c-5p	-0.07	0.96484	1.627595229	11991.65	14107.32
260.hsa-miR-218-5p	0.07	0.96679	1.656090359	11145.78	11277.41
261.hsa-miR-98-5p	-0.06	0.96914	1.646061408	1998.216	2224.64
262.hsa-miR-30d-5p	0.06	0.96915	1.651907743	17656.26	17970.46
263.hsa-let-7i-5p	0.06	0.97000	1.6230589	23889.9	25451.54
264.hsa-miR-378h	0.15	0.97012	3.969471452	0.66	0.53

265.hsa-miR-103a-2-5p	0.12	0.97013	3.267670489	16.67	14.14
266.hsa-miR-194-5p	-0.09	0.97092	2.589049921	476.99	501.33
267.hsa-miR-1185-5p	0.11	0.97114	2.921922883	56.32	54.55
268.hsa-miR-3059-5p	0.09	0.97123	2.535828059	57.98	55.30
269.hsa-miR-100-5p	-0.05	0.97322	1.631593062	26410.55	31307.8
270.hsa-miR-338-5p	0.05	0.97611	1.699196774	3024.208	3536.43
271.hsa-miR-26a-5p	-0.05	0.97766	1.62643163	105819.7	118492.3
272.hsa-miR-873-5p	0.07	0.97831	2.537224159	83.96	82.08
273.hsa-miR-21-5p	0.04	0.97852	1.627358017	18532.3	19704.91
274.hsa-miR-376a-5p	0.06	0.97946	2.300398267	739.73	307.07
275.hsa-miR-1298-5p	0.06	0.98158	2.575003903	106.77	101.26
276.hsa-miR-335-5p	0.04	0.98382	2.178833818	607.15	650.82
277.hsa-miR-488-5p	0.07	0.98406	3.740758473	11.29	10.49
278.hsa-miR-191-5p	0.03	0.98714	1.635515147	9085.61	10238.19
279.hsa-miR-378i	-0.04	0.98721	2.533250803	166.34	168.26
280.hsa-miR-29c-5p	-0.04	0.98784	2.548146052	212.38	217.50
281.hsa-miR-3613-5p	0.04	0.98882	2.548246305	47.67	46.25
282.hsa-miR-199b-5p	-0.03	0.98962	2.537614201	114.13	115.10
283.hsa-miR-9-5p	-0.02	0.99028	1.626858462	130150.1	143311.6
284.hsa-miR-497-5p	0.03	0.99029	2.565124265	316.28	306.00
285.hsa-miR-99b-5p	-0.02	0.99238	1.633016537	9702.66	11185.79
286.hsa-miR-195-5p	-0.02	0.99290	1.694882639	559.45	610.88
287.hsa-miR-486-5p	-0.01	0.99667	2.529567144	158.19	157.23
288.hsa-miR-145-5p	0.01	0.99749	2.308261187	1580.13	1579.11
289.hsa-let-7b-5p	0.48	N/A	1.892881953	56644.28	52856.15

Supplementary Table 2. Complete list of piRNAs expressed in EVs control and upon CSC exposure.

piRNAs	Log2 Fold Change	*p* value	lfcSE	Read Count (Avg)	
				CSC EVs	Control EVs
1. piR-50603	9.78	0.001	3.16	276.51	0
2. piR-36705	- 10.19	0.002	3.31	0	191.02
3. piR-37183	- 10.19	0.002	3.31	0	191.02
4. piR-59260	- 10.19	0.002	3.31	0	191.02
5. piR-36924	- 10.24	0.002	3.40	0.32	358.4
6. piR-31985	10.52	0.004	3.69	498.48	0
7. piR-52900	- 10.28	0.004	3.65	0	211.63
8. piR-32679	0.61	0.8	2.46	3239.06	2504.47
9. piR-33043	1.33	0.5	2.25	46035.21	14844.69
10. piR-33044	0.89	0.69	2.3	11972.82	4910.253
11. piR-33065	0.35	0.88	2.41	2125.942	2095.11
12. piR-33151	0.32	0.88	2.28	18916.44	17546.12
13. piR-33161	0.15	0.94	2.34	1261.04	1383.33
14. piR-33437	- 1.39	0.55	2.34	131.37	426.00
15. piR-33468	0.62	0.81	2.7	14053.01	10994.76
16. piR-35284	0.45	0.85	2.51	23541.48	20444.46
17. piR-35413	-0.48	0.82	2.23	52580.15	75646.9
18. piR-35463	- 1.16	N/A	2.23	5583.8	10974.3
19. piR-35469	0.11	0.96	2.44	5230.71	5720.52
20. piR-35982	0.37	0.87	2.46	62480.44	57048.11
21. piR-36034	- 1.42	0.59	2.65	1497.51	2978.34
22. piR-36036	- 1.42	0.59	2.65	1498.03	2977.82
23. piR-36037	- 1.35	0.60	2.64	1692.43	3244.99
24. piR-36041	0.09	0.96	2.32	3875.85	4244.99
25. piR-36063	- 0.43	0.85	2.39	2600.82	2923.09
26. piR-36074	- 0.11	0.95	2.21	343.40	356.16
27. piR-36082	- 1.33	0.59	2.53	330.39	658.90
28. piR-36170	1.33	0.58	2.27	895.83	282.14
29. piR-36241	- 1.50	0.51	2.31	498.45	1181.02
30. piR-36243	- 1.12	0.61	2.24	378.35	689.09
31. piR-36246	- 1.36	0.56	2.34	3830.48	3
32. piR-36249	- 1.29	0.57	2.33	706.13	1580.22

33. piR-36256	- 0.06	0.97	2.27	675.60	726.18
34. piR-36378	- 1.54	0.49	2.25	436.60	1116.14
35. piR-36511	- 0.64	0.77	2.25	1612.06	2062.3
36. piR-36678	1.33	0.61	2.62	1288.57	419.50
37. piR-36743	0.10	0.96	2.27	13445.55	13101.68
38. piR-43770	1.14	N/A	2.31	944.24	304.86
39. piR-43772	0.55	0.80	2.23	87706.92	59668.2
40. piR-43768	- 1.57	0.49	2.30	4.34	11.27
41. piR-44312	- 1.34	0.59	2.50	216.88	595.70
42. piR-44984	0.01	0.99	2.32	3444.76	4009.44
43. piR-44992	- 1.09	0.66	2.57	704.04	1168.54
44. piR-45029	- 0.95	0.75	3.06	1142.22	1753.99
45. piR-46086	- 3.41	N/A	2.79	25.71	217.48
46. piR-46895	0.63	0.77	2.25	1104.97	577.46
47. piR-49143	- 0.20	0.92	2.23	85341.22	93742.34
48. piR-49144	- 0.11	0.96	2.21	106069.9	105445.8
49. piR-49145	- 0.14	0.94	2.24	86819.11	84319.73
50. piR-50176	- 1.8	0.52	2.86	602.27	1599.04
51. piR-52404	- 0.37	N/A	2.58	566.70	725.65
52. piR-52882	- 1.03	0.70	2.69	1906.75	3286.76
53. piR-57516	1.23	0.58	2.25	401.85	141.00
54. piR-57660	- 0.14	0.94	2.23	5545.42	550.46
55. piR-57942	0.02	0.99	2.55	258.82	305.17
56. piR-60565	0.46	0.83	2.25	20433.12	16355.46
57. piR-61645	0.31	0.9	2.5	12449.54	11836.3
58. piR-61648	- 0.21	0.93	2.53	31325.73	41210.36
59. piR-61651	1.19	0.59	2.25	339.46	124.47
60. piR-62011	1.53	0.5	2.28	1161.90	310.88
61. piR-31924	0.31	0.9	2.57	28339.52	26634.57
62. piR-31925	0.21	0.93	2.59	53376.28	54198.95
